# Morphometric analysis and immunobiological investigation of *Indigofera oblongifolia* on the infected lung with *Plasmodium chabaudi*

**DOI:** 10.1515/biol-2025-1110

**Published:** 2025-08-20

**Authors:** Mutee Murshed, Jameel Al-Tamimi, Hossam Ebaid, Saleh Al-Quraishy

**Affiliations:** Department of Zoology, College of Science, King Saud University, P.O. Box 2455, Riyadh, 11451, Saudi Arabia

**Keywords:** lung inflammation, *Indigofera oblongifolia*, *Plasmodium chabaudi*, TNF-a, IL-6, immunobiological

## Abstract

The present study aimed to evaluate the therapeutic potential of *Indigofera oblongifolia* with silver nanoparticles (AgNPs) and chloroquine (CQ) 10 mg/kg in treating lung inflammation caused by *Plasmodium chabaudi* infection in a mouse model. Fifty female C57BL/6 mice were divided into five groups: control, *Indigofera oblongifolia* leaf extract (IOLE) AgNPs treated, *P. chabaudi* infected, infected and IOLE AgNPs treated, infected and CQ 10 mg/kg treated. Lung histopathology was assessed using microscopic analysis and immunohistochemistry investigation for TNF-α and IL-6. The results showed that the positive control of AgNPs slightly triggered proinflammatory cytokines and created an oxidative stress status in lung tissue. The group IOLE AgNPs treatment significantly restored the normal organization of the control lung tissue. It reduced alveolar and septal congestion, edema, and necrosis compared to the infected lung. Therefore *I. oblongifolia* as a natural medical plant displayed significant antimalarial and anti-oxidant properties effectively, reducing inflammatory signs and cytokine levels in *P. chabaudi*-infected lungs and treating the harmful impact of AgNPs in *P. chabaudi*-infected + *I. oblongifolia* with AgNPs lung. While CQ shows limited efficiency, it showed moderate improvement in the histological architecture such as thicker alveolar and bronchiolar walls and restricted expansion. However, the septal and alveolar congestion, hemosiderin concentration, edema, and necrotic cells were still present. Also, immunohistochemistry expression of proinflammatory cytokines is still expressed. In conclusion, this study highlights the therapeutic potential of *I. oblongifolia* for malaria management. Also, this study uniquely explored the combined influences of *I. oblongifolia* leaf extract and AgNPs on lung inflammation caused by *P. chabaudi* infection. Previous studies may have explored these components separately, but the current study examines their synergistic potential in treating malaria-related lung pathology. Consequently, the study compared the efficacy of *I. oblongifolia* with that of CQ, revealing that the latter exhibited limited efficiency due to drug resistance and its inability to restore the normal features of its histology. This comparison highlights the potential impact *of I. oblongifolia* as a more effective alternative in malaria treatment, particularly in cases where conventional drugs fail.

## Graphical abstract

**Figure:**
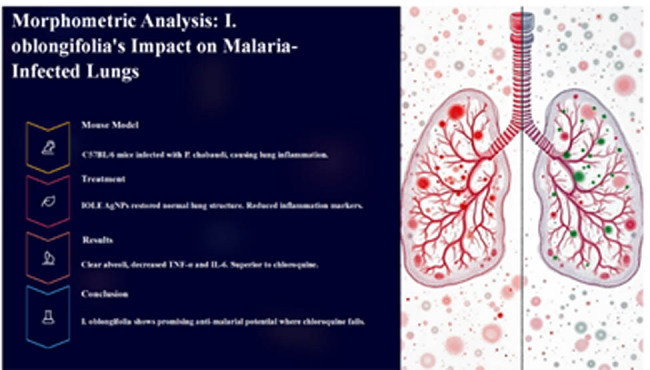

## 1 Introduction

Malaria, caused by parasites of the genus *Plasmodium*, remains a global health concern, with *Plasmodium chabaudi* serving as a model for studying the pathogenesis of this disease in mice [1]. This parasite is known to sequester predominantly in the liver and lungs, leading to organ-specific pathology in these organs [2]. The lung is a vital organ in pulmonary circulation, essential for gas exchange and converting deoxygenated blood into oxygenated blood [3]. The lung experiences notable histopathological changes during infection, including increased infiltration of immune cells and cellularity of the alveolar septae [4]. Malaria is widely recognized as the most common epidemic disease globally [5].

Despite the well-documented pathogenesis of malaria-induced lung pathology, there is growing interest in exploring novel therapeutic approaches to mitigate these effects [6]. Recent studies have focused on exploring novel therapeutic approaches to combat malaria and its associated complications [7]. Medical plants have been integral to human history and have diverse therapeutic properties in treating serious diseases [8]. Compounds produced from plants have become viable options for treating malaria, and *Indigofera oblongifolia* is a promising example of this [9]. *I. oblongifolia* is a plant that belongs to the Fabaceae family [10]. One such approach involves the use of plant-derived compounds, specifically *Indigofera oblongifolia* leaf extract (IOLE) silver nanoparticles (AgNPs). These nanoparticles have shown promise in mitigating the effects of *P. chabaudi* infection in the kidneys [11].

Cytokines are essential in the immunological response to malaria infection [12]. Tumor necrosis factor-alpha (TNF-α) and interleukin-6 (IL-6) [13] are important in the pathogenesis of malaria-induced lung pathology [14]. Elevated levels of these cytokines are associated with increased disease severity and mortality in *P. chabaudi* infections [13]. The modulation of TNF-α and IL-6 by potential therapeutic agents is of significant interest in developing effective treatments for malaria-associated complications.

This study aims to provide a morphometric analysis and immunobiological investigation of the effects of *I. oblongifolia* leaf extract with AgNPs on lung tissue infected with *P. chabaudi*. We aim to understand the structural changes in lung tissue following the administration of IOLE AgNPs and investigate how these changes could aid in the fight against malaria through the use of immunobiological techniques and standard histological methods.

## 2 Materials and methods

### 2.1 *In vivo* in the mice and parasites

Fifty female C57BL/6 mice, weighing 22 ± 3 g and 9 ± 2 weeks old, were supplied by the King Faisal Hospital Research Unit in Riyadh, Saudi Arabia. We acquired the *P. chabaudi* strain from the parasitology laboratory at King Saud University’s College of Science. We then transferred *P. chabaudi* cryopreserved parasites into uninfected mice. A 100 μL PBS solution containing 105 parasitized erythrocytes was delivered intraperitoneally (i.p.) to the mouse.

Five distinct categories were assigned to the animals. Ten mice were included in each of the groups. The first control group that was not infected received PBS by oral administration for 7 days. IOLE AgNPs were given orally to the second group at a dose of 50 mg/kg daily for 7 days. Fifty female C57BL/6 mice, weighing 22 ± 3 g and 9 ± 2 weeks old, were supplied by the King Faisal Hospital Research Unit in Riyadh, Saudi Arabia. One hour later, the fourth group got 50 mg/kg of IOLE AgNPs orally for 7 days, while the fifth group received 10 mg/kg chloroquine (CQ) phosphate (Sigma-Aldrich, St. Louis, MO) orally for 4 days.


**Ethical approval:** The research related to animal use has been complied with all the relevant national regulations and institutional policies for the care and use of animals, and has been approved by the Ethics Committee of King Saud University (approval number: KSU-SE-21-86).

### 2.2 Biosynthesized AgNPs

In the literature, a procedure for making the extract is described. Following the methodology described in a previous study, the preparation of AgNPs was carried out.

## 3 Immunobiological assessment

### 3.1 Tissue collection and processing

On the seventh day following infection, all mice were euthanized by CO_2_ asphyxiation and then dissected to collect lung tissues. The lung was dissected and sectioned for pathological examination. The tiny fragments were immersed in a solution of neutral buffered formalin (10%) for histological analysis. On Day 8 post-infection, mice were euthanized by CO_2_, and lungs were harvested. The lung was fixed in 10% neutral buffered formalin for histological analysis.

### 3.2 Lung immunohistopathology

Immunohistochemical staining was performed using antibodies against TNF-α and IL-6. The stained sections were analyzed using image analysis software ImageJ (Version 1.54k) according to the protocols in the literature to quantify TNF-α and IL-6 levels in lung tissue.

### 3.3 Statistical analysis

Data were analyzed using GraphPad Prism 5. We used a one-way ANOVA and Tukey’s *post-hoc* test for multiple comparisons. Results are expressed as mean ± SEM. *p*-Values <0.05 were considered statistically significant.

## 4 Results

In the current investigation, the pharmacological activity of IOLE AgNPs was studied as a potential therapy for inflammation of the lungs caused by *P. chabaudi*, which is a parasite that causes malaria sickness. The study investigated the morphometric measurements in addition to the histopathological characteristics of five groups: the control lung, the lung that had been treated with IOLE AgNPs, the lung that had been infected with *P. chabaudi*, the lung that had been treated with IOLE AgNPs after inflammation with *P. chabaudi*, and finally, the lung that had been treated with CQ 10 mg/kg after inflammation with *P. chabaudi*. Positive control of IOLE AgNPs and the lung treated with it showed a considerable improvement in the histoarchitecture of lung tissue, as demonstrated by the current experiment.

### 4.1 Microscopic examination

The microscopic analysis of five experimental groups revealed that the impact of IOLE AgNPs on lung tissue expressed a significant (*p* < 0.05) expansion of both bronchiolar (701.70 ± 5.08 µm) and alveolar (214.62 ± 1.33 µm) walls compared to the control ducts. However, the thickness of their walls was reduced (15.78 ± 0.50 µm) and (7.81 ± 0.58 µm), respectively, in a significant way (*p <* 0.05). Additionally, the diameter of interstitial tissue (219.06 ± 1.63 µm), the intensity of septal (2.78 ± 0.13%), and alveolar (1.64 ± 0.08%) congestion and edema (2.84 ± 0.44%) elevated non-significantly (*p* > 0.05) compared to those in the normal lung. However, the hemosiderin (4.00 ± 0.10%) and necrosis (1.92 ± 0.02%) intensity increased significantly (*p* < 0.05). Alternatively, the infected lung with *P. chabaudi* displayed significant (*p* < 0.001) restriction in the cavity of pulmonary ducts such as alveolar (58.83 ± 1.37 µm) and bronchiolar (105.69 ± 3.78 µm) ducts comparatively less than the control ducts, and also significant (*p* < 0.001) thickness in alveolar wall (31.32 ± 0.72 µm) and non-significant (*p* > 0.05) increase in the thickness of bronchiolar wall (21.26 ± 0.94 µm) in comparison to the control thickness. The *P. chabaudi* eads to lung severance of increasing intensity (*p* < 0.001) of pathological inflammatory signs such as septal congestion (35.66 ± 1.39%), alveolar congestion (69.42 ± 2.72%), edema (42.53 ± 1.70%), hemosiderin intensity (20.57 ± 0.6%), and necrosis intensity (14.84 ± 0.6%) more than the normal ranges of these signs in control lung. Therefore, the interstitial tissue expressed an increase in the diameter higher than the normal ones (147.84 ± 3.44 µm) as shown in Table 1.

**Table 1:** Morphometric analysis of histopathological parameters of lung tissue: (a) alveolar expansion, (b) alveolar wall thickness, (c) bronchiolar dimensions, (d) bronchiolar wall thickness, (e) hemosiderin intensity, (f) diameter of the interstitium, (g) alveolar congestion, (h) septal congestion, (i) intensity of necrosis, and (j) edema

	Layers									
Groups	Alveolar expansion (µm)	Alveolar wall thickness (µm)	Bronchiolar wall thickness (µm)	Bronchiolar dimensions (µm)	Interstitial diameter (µm)	Septal congestion (%)	Alveolar congestion (%)	Edema (%)	Hemosiderin (%)	Necrosis intensity (%)
Control	200.47 ± 2.54	10.82 ± 0.46	19.60 ± 0.27	581.68 ± 6.22	128.40 ± 2.65	0.17 ± 0.01	0.09 ± 0.00	0.21 ± 0.01	1.28 ± 0.15	0.48 ± 0.04
IOLEAgNPs	214.62 ± 1.33*	7.81 ± 0.58	15.78 ± 0.50*	701.70 ± 5.08***	219.06 ± 1.63**	2.78 ± 0.13	1.64 ± 0.08	2.84 ± 0.44	4.00 ± 0.10*	1.92 ± 0.02*
Infected	58.83 ± 1.37	31.32 ± 0.72***	21.26 ± 0.94**	105.69 ± 3.78	305.69 ± 3.78***	35.66 ± 1.39***	69.42 ± 2.72**	42.53 ± 1.70***	20.57 ± 0.6***	14.84 ± 0.6***
Infected + IOLEAgNPs	202.91 ± 2.69**	18.64 ± 0.85**	16.84 ± 0.75*	499.69 ± 8.58**	147.84 ± 3.44*	7.84 ± 0.73*	1.84 ± 0.12	9.09 ± 0.24*	4.13 ± 0.12*	1.97 ± 0.14*
Infected + CQ10	182.47 ± 0.87*	22.40 ± 0.28***	20.69 ± 1.22**	396.62 ± 4.44*	236.48 ± 2.08***	17.66 ± 0.62**	18.93 ± 0.4*	25.30 ± 1.77**	6.74 ± 0.30**	4.48 ± 0.15**

Among five groups: the control, *I. oblongifolia* with silver nanoparticles (IOLE AgNPs) group, the group infected by *P. chabaudi*, the group infected by *P. chabaudi* and 50 mg/kg of IOLE AgNPs, and the group administrated with *P. chabaudi* and CQ10. Values are represented as mean ± STD & *n* = 10 animals. Mean values within the same parameter and not sharing a common superscript symbol(s) differ significantly at *p < 0.05, **p < 0.01, ***p < 0.001, and values that are recorded with the non-significance difference (n.s.) and *p*-value was calculated according to comparison with the control group.

After treatment with 50 mg/kg of IOLE AgNPs after inflammation with *P. chabaudi*, the pulmonary ducts displayed a significant recovery compared to the normal dimensions and thickness, and recorded a non-significant (*p* > 0.05) diameter in alveolar (202.91 ± 2.69 µm) and bronchiolar (499.69 ± 8.58 µm) ducts compared to the control. All pathological inflammatory signs exhibited a significant (*p* < 0.05) reduction in their intensity in this group. However, the CQ 10 mg/kg therapy after inflammation with *P. chabaudi* revealed a slow recovery compared to the normal histological architecture, while the alveolar (182.47 ± 0.87 µm) and bronchiolar (396.62 ± 4.44 µm) ducts still had a restricted cavity less than the control diameter. Also, the alveolar wall (22.40 ± 0.28 µm) significantly thickened (*p* < 0.05) and the bronchiolar wall showed non-significant (*p* > 0.05) thickness (20.69 ± 1.22 µm). Furthermore, all the pathological inflammatory signs such as septal congestion (17.66 ± 0.62%), alveolar congestion (18.93 ± 0.4%), edema (25.30 ± 1.77%), hemosiderin intensity (6.74 ± 0.30%), and necrosis intensity (4.48 ± 0.15%) still elevated in comparison to normal ranges of these signs in the control lung as shown in Table 1.

## 5 Immunohistochemistry investigation

### 5.1 TNF-α assay

**Figure 1:**
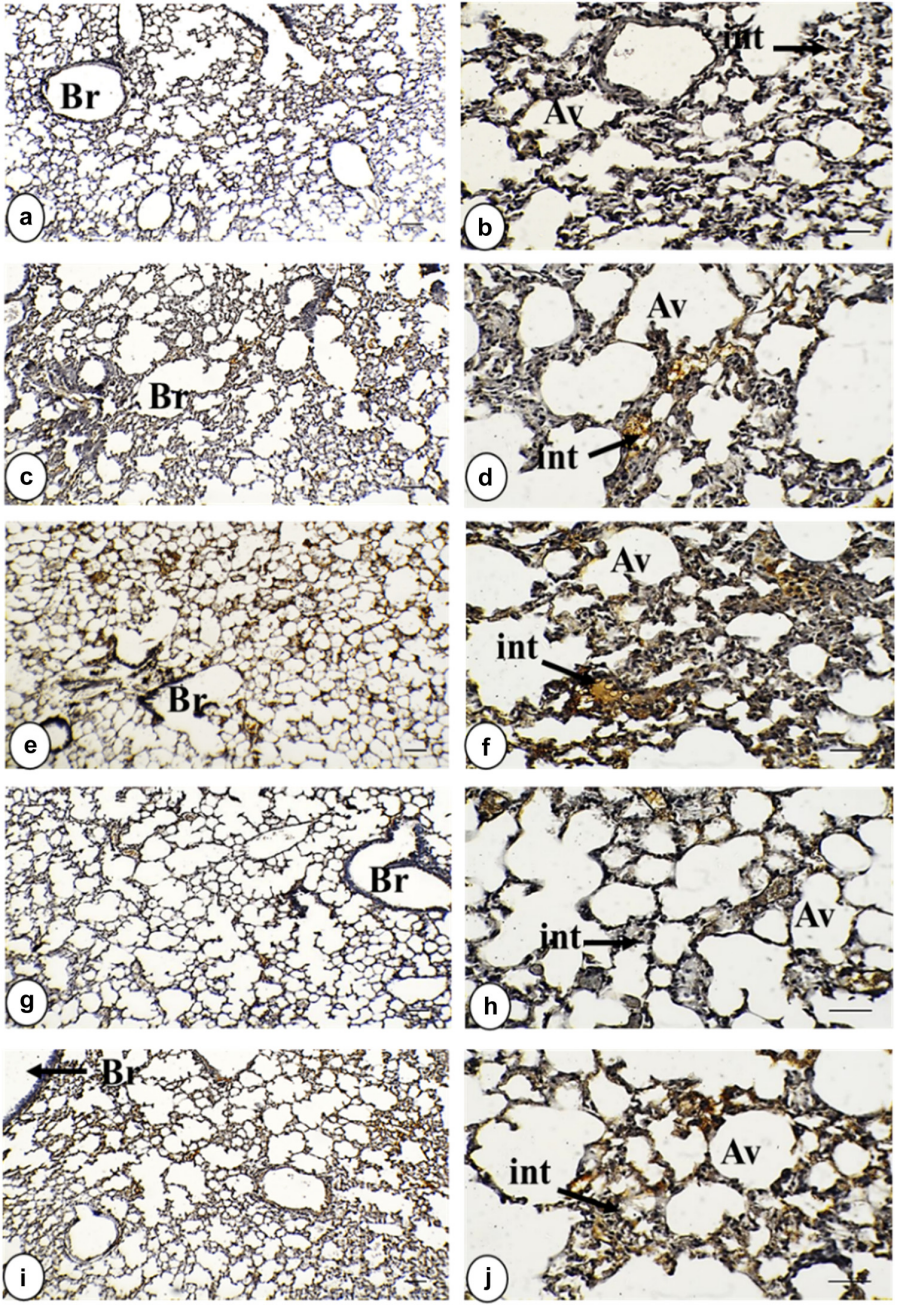
Photomicrograph of TNF-α-stained lung tissue in all study groups. (a) and (b) Control group displayed a negative expression of TNF-α within the interstitial tissue, bronchiolar wall, and alveolar wall. (c) and (d) Group of IOLE AgNPs exhibited mild positive expression of TNF-α within the interstitial tissue in the form of yellowish spots. (e) and (f) Infected group shows a strong positive expression of TNF-α in the form of brownish patches in the interstitial tissue, bronchiolar wall, and alveolar wall. (g) and (h) Group infected + 50 mg/kg IOLE AgNPs expressed low positive staining of TNF-α within the interstitial tissue in the form of yellowish spots. (i) and (j) Group with CQ 10 mg/kg presented mild positive expression of TNF-α, within the interstitial tissue and alveolar wall in the form of yellowish patches (TNF-α ×, a, c, e, g, and i = 100 µm and b, d, f, h, and j = 400 µm). TNF-α expression is indicated by black arrows. Stained by DAB-chromogen (brown color) immunostaining.

The immunohistochemical sections did not reveal the reactivity of the anti-TNF-α antibody immunostaining in the control lung. Administration of IOLE AgNPs showed a few positive yellowish spots dispersed over the interstitial areas with a significant (*p* < 0.05) intensity (3.05 ± 0.18%) and around the alveolar wall (1.65 ± 0.18%). However, *Plasmodium* infection caused intensive (*p* < 0.01) immunostaining around the bronchiolar (5.78 ± 0.54%) and alveolar wall (2.50 ± 0.24%) and interstitial area (13.35 ± 0.43%) compared to the control intensities. The lung treated with IOLE AgNPs still displayed a significant (*p* < 0.01) positive interaction with TNF-α immunostaining in the interstitial area (4.56 ± 0.34%), while both bronchiolar (1.80 ± 0.09%) and alveolar (0.67 ± 0.08%) walls exhibited non-significant (*p* > 0.05) intensities compared to the control tissue. However, yellow patches were still significantly observed (*p* < 0.05) around the infected + CQ10 bronchiolar wall (4.04 ± 0.23%) and the alveolar wall (1.60 ± 0.23%), but the interstitial tissue displayed higher significant intensity (*p* < 0.001) in this group (10.18 ± 0.38%) (Figures 1a–j and 3a and Table 2).

**Table 2:** TNF expression immunohistochemistry in lung tissue stained by DAB-chromogen within a bronchiolar wall, alveolar wall, and interstitial tissue among the five groups

Groups	Layers		
	Bronchiolar wall	Alveolar wall	Interstitial tissue
Control	0.76 ± 0.09	0.26 ± 0.04	1.08 ± 0.07
IOLE AgNPs	1.78 ± 0.06*	1.65 ± 0.18*	3.05 ± 0.18*
Infected	5.78 ± 0.541**	2.50 ± 0.242**	13.35 ± 0.43**
Infected + IOLE AgNPs	1.80 ± 0.09	0.67 ± 0.08	4.56 ± 0.34**
Infected + CQ10	4.04 ± 0.23*	1.60 ± 0.23*	10.18 ± 0.38**

Control, IOLE AgNPs group, group infected by *P. chabaudi*, group infected by *P. chabaudi* and 50 mg/kg of IOLE AgNPs, and the group administrated with *P. chabaudi* and CQ 10 mg/kg. Values are represented as mean ± STD & *n* = 10 animals. Mean values within the same parameter and not sharing a common superscript symbol(s) differ significantly at *p < 0.05, **p < 0.01, ***p < 0.001, and values that are recorded with a non-significance difference (n.s.) and *p*-value was calculated according to comparison with the control group.

### 5.2 IL-6 assay

**Figure 2:**
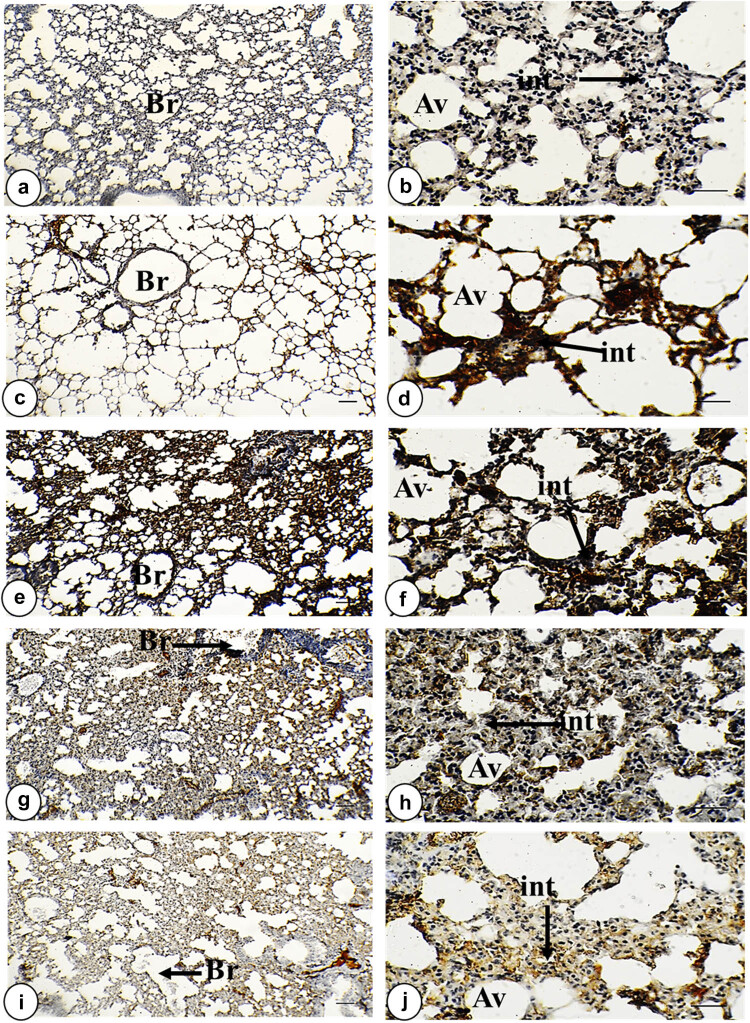
Photomicrograph of IL-6-stained lung tissue in all study groups. (a) and (b) Control group exhibited a negative expression of IL-6 within the interstitial tissue bronchiolar wall and alveolar wall. (c) and (d) Group of IOLE AgNPs shows a moderate positive expression of IL-6 within the interstitial tissue and alveolar wall as brownish spots. (e) and (f) Infected group displayed strong positive staining of IL-6 within the interstitial tissue, bronchiolar wall, and alveolar wall as brownish patches. (g) and (h) Group with infected + 50 mg/kg IOLE AgNPs presented mild positive expression of IL-6 within the interstitial tissue and alveolar wall as brownish spots. (i) and (j) Group with infected + CQ 10 mg/kg shows a higher positive expression of IL-6 within the interstitial tissue and alveolar wall as yellowish patches (IL-6 × a, c, e, g, and i = 100 µm and b, d, f, h, and j = 400 µm). IL-6 expression is indicated by black arrows. Stained by DAB-chromogen (brown color) immunostaining.

**Figure 3:**
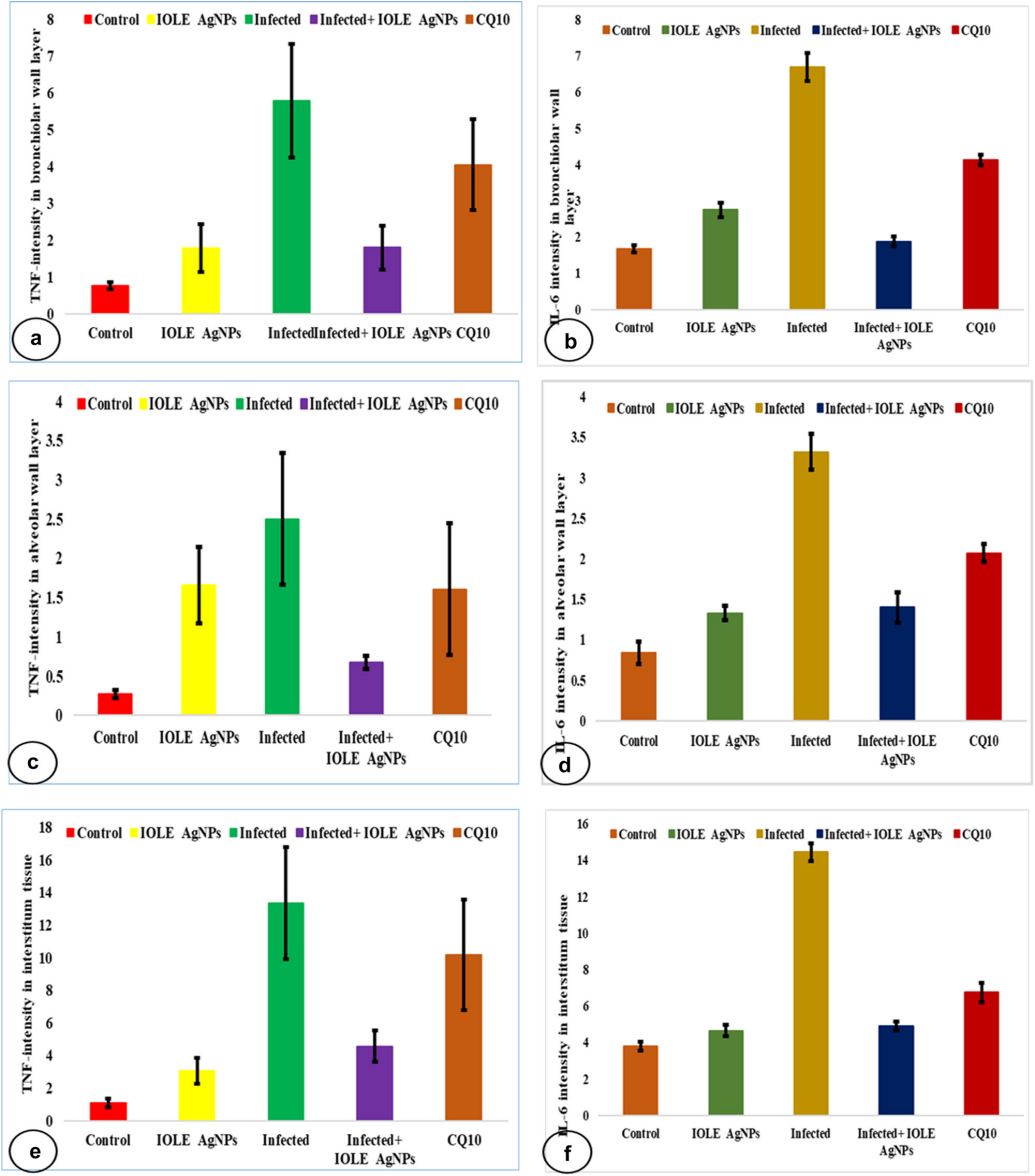
Bar graphs of immunohistochemistry showing (a), (c), (e) TNF expression and (b), (d), (f) IL-6 expression in lung tissue stained by DAB-chromogen (brown color) within a bronchiolar wall (a and b), alveolar wall (c) and (d), and interstitial tissue (e) and (f). Values are represented as mean ± STD & *n* = 10 animals. Mean values within the same parameter and not sharing a common superscript symbol(s) differ significantly at *p* < 0.05, and values that are recorded with non-significance difference (n.s.).

The anti-IL-6 antibody immunostaining did not react with any antigen in the control lung. Sections taken from the IOLE AgNPs treated group showed slight upregulation in the anti-IL-6 antibodies (*p* > 0.05) within the bronchiolar wall (2.75 ± 0.20%) in the form of brownish spots and non-significant expression (*p* > 0.05) in alveolar wall (1.33 ± 0.09%) and interstitial tissue (4.65 ± 0.30%) compared to control sections. However, a strong positive staining of IL-6 was observed in the bronchiolar wall (6.70 ± 0.39%), alveolar wall (3.32 ± 0.22%), and interstitial tissue (14.44 ± 0.48%) of the plasmodium-infected sections in comparison with normal tissue. The treatment sections of the *I. oblongifolia* displayed low positive expression of IL-6 in the bronchiolar wall (1.88 ± 0.14%) and alveolar wall (1.40 ± 0.19%) in the form of brownish spots and non-significant expression (*p* > 0.05) with control sections in the interstitial tissue (4.89 ± 0.25%). On the contrary, the group with infected + CQ 10 mg/kg showed positive staining (*p* < 0.01) of IL-6 in the bronchiolar wall (4.13 ± 0.15%), and moderate expression (*p* < 0.05) in the alveolar wall (2.07 ± 0.11%) and the interstitial tissue (6.74 ± 0.52%) in the form of yellowish patches. Our findings of a semi-quantitative analysis of immunostaining for TNF-α and IL-6 proinflammatory cytokines are illustrated in Figures 2a–j and 3b and Table 3.

**Table 3:** IL-6 expression immunohistochemistry in lung tissue stained by DAB-chromogen within a bronchiolar wall, alveolar wall, and interstitial tissue among the five groups

	Layers		
Groups	Bronchiolar wall	Alveolar wall	Interstitial tissue
Control	1.68 ± 0.10	0.84 ± 0.14	3.79 ± 0.26
IOLE AgNPs	2.75 ± 0.20*	1.33 ± 0.09	4.65 ± 0.30
Infected	6.70 ± 0.39***	3.32 ± 0.22**	14.44 ± 0.48**
Infected + IOLE AgNPs	1.88 ± 0.14*	1.40 ± 0.19*	4.89 ± 0.25*
Infected + CQ10	4.13 ± 0.15**	2.07 ± 0.11*	6.74 ± 0.52*

Control, IOLE AgNPs group, group infected by *P. chabaudi*, group infected by *P. chabaudi* and 50 mg/kg of IOLE AgNPs, and the group administrated with *P. chabaudi* and CQ 10 mg/kg. Values are represented as mean ± STD & *n* = 10 animals. Mean values within the same parameter and not sharing a common superscript symbol(s) differ significantly at *p < 0.05, **p < 0.01, ***p < 0.001, and values that are recorded with a non-significance difference (n.s.) and *p*-value was calculated according to comparison with the control group.

### 5.3 Correlation analysis

**Figure 4:**
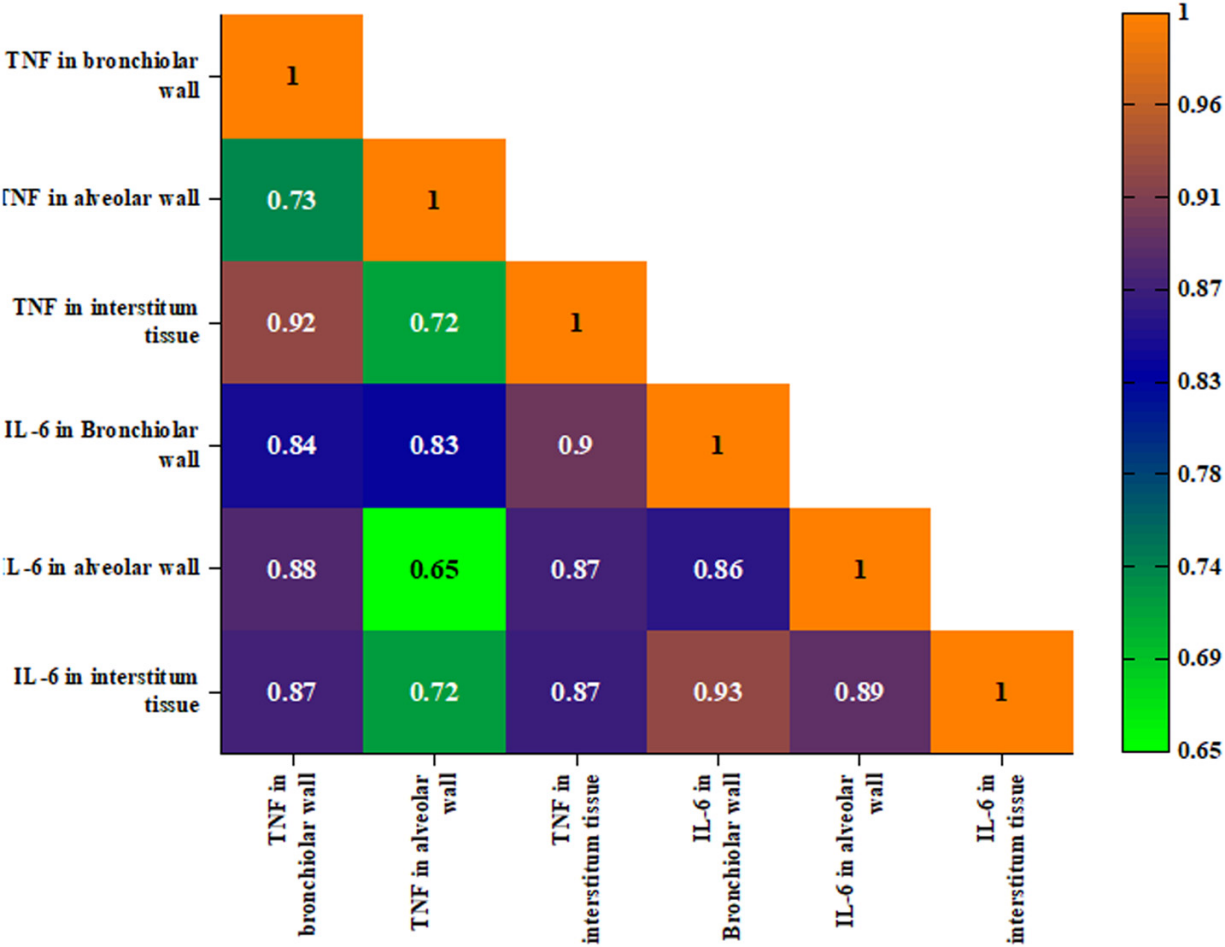
Triangle heatmap representing Pearson correlation coefficients between TNF-α and IL-6 expression immunohistochemistry in lung tissue stained by DAB-chromogen within a bronchiolar wall, alveolar wall, and interstitial tissue among five groups: control, IOLE AgNPs-infected, infected, infected + 50 mg/kg IOLE AgNPs, and CQ10-treated. The strength and direction of correlations: positive correlations are represented in purple color, while negative correlations are in orange color. The intensity of the color reflects the strength of the correlation, with darker shades indicating stronger associations.

The heatmap of TNF-α and IL-6 expression immunohistochemistry in lung regions (bronchiolar wall, alveolar wall, and interstitial regions) indicated strong coordination in the inflammatory response whereas the findings revealed a strong correlation between TNF-α and IL-6 expressions in three different regions in lung tissues. For instance, TNF-α expression in the bronchiolar wall was positively correlated with alveolar wall expression (*r* = 0.733, *p* < 0.01) and interstitial expression (*r* = 0.924, *p* < 0.01) suggesting that the inflammatory response was coordinated all over the whole tissue and not in a single region. Similarly, IL-6 expression in the bronchiolar wall was strongly correlated with its expression in the alveolar wall (*r* = 0.855, *p* < 0.01) and interstitial expression (*r* = 0.926, *p* < 0.01), indicating similar coordination in the inflammatory response in the whole regions of the organ. For cross-cytokine relationships, the heatmap exhibited high inter-cytokines correlation among the investigated regions, example.g., the TNF-α expressions in the bronchiolar wall correlated strongly with the expression of IL-6 in the bronchiolar wall (*r* = 0.841, *p* < 0.01), alveolar wall (*r* = 0.879, *p* < 0.01), and interstitium (*r* = 0.869, *p* < 0.01), which indicates that the increase in the TNF-α expression was accompanied by the increase in IL-6 suggesting that there was coordination across the inflammatory response from the beginning of the inflammation to the beginning of the recovery (Table 4 and Figure 4).

**Table 4:** Pearson correlation matrix of TNF-α and IL-6 expression immunohistochemistry in lung tissue stained by DAB-chromogen within a bronchiolar wall, alveolar wall, and interstitial tissue among five groups: control, IOLE AgNPs-infected, infected, infected + 50 mg/kg IOLE AgNPs, and CQ10-treated

Correlated parameter	TNF in bronchiolar wall	TNF in alveolar wall	TNF in interstitial tissue	IL-6 in bronchiolar wall	IL-6 in alveolar wall	IL-6 in interstitial tissue
TNF in bronchiolar wall	1	0.733**	0.924**	0.841**	0.879**	0.869**
TNF in alveolar wall	0.733**	1	0.717**	0.831**	0.650**	0.722**
TNFin interstitial tissue	0.924**	0.717**	1	0.898**	0.870**	0.868**
IL-6 in bronchiolar wall	0.841**	0.831**	0.898**	1	0.855**	0.926**
IL-6 in alveolar wall	0.879**	0.650**	0.870**	0.855**	1	0.889**
IL-6 in interstitial tissue	0.869**	0.722**	0.868**	0.926**	0.889**	1

The heatmap displays the strength and direction of correlations and statistical significance is denoted as *p* < 0.05 (*), *p* < 0.01 (**).

## 6 Discussion

*P. chabaudi* is considered a model of the malaria parasite to explore its vigorous impact on different organs such as the lungs. It is known that malaria disease induces multiple inflammatory signs besides several cytokines like TNF-α and IL-6 playing a significant role in the inflammatory process. Therefore, the present study indicates the potential impact of *I. oblongifolia* as an effective therapy for malaria disease, focusing on the microscopic analysis and immunobiological alterations particularly the levels of TNF-α and IL-6.

The current investigation showed that the extraction of *I. oblongifolia* leaf had significant antimalarial and anti-oxidant properties that could reduce the inflammatory signs caused by *P. chabaudi* infection via the modulation of inflammatory cytokines and preventing oxidative stress in the infected lung. The present analysis of the study displayed slight restriction in the pulmonary ducts with a thicker wall than the normal thickness in the positive control group of *I. oblongifolia* with AgNPs. Also, dispersion of necrotic cells and septal congestion besides small spots of edema were observed. De Matteis [15] and Singh et al. [16] explained that these findings are due to the repeated exposure to AgNPs, even at low doses, resulting in cumulative impacts that can lead to chronic inflammation and fibrosis. Consequently, the accumulation of AgNPs in the bronchiolar and alveolar ducts aids in triggering oxidative stress status and inflammation. Several studies validated the previously reported findings of AgNPs, including glomerulosclerosis and necrosis in the kidney [17,18], congestion of white pulp in the spleen [19,20], cardiac ischemic reperfusion injury in the heart [21–23], apoptosis in the thymus [24], and agyria in the skin [25–27]. Also, the present study revealed that the pro-inflammatory cytokines TNF-α and IL-6 assay displayed non-significant expression compared to normal reactions around bronchiolar and alveolar walls while significant yellowish spots in the interstitial tissue. Similar findings were recorded by Alessandrini et al. [28] and Pitchai et al. [29].

The lung infected with *P. chabaudi* registered a significant obstruction in bronchiolar and alveolar ducts, in addition to elevated rates of alveolar and septal congestion, edema, hemosiderin, and necrosis intensity. Brugat et al. and Nahrendorf et al. [2,30] found a significant increase in dendritic, neutrophil, and macrophage density during acute lung infection with *P. chabaudi*, as detected by flow cytometry. Moreover, severe cytotoxicity was demonstrated in many organs such as the liver, kidney, spleen, and brain during acute inflammation with malaria [31–33]. Acute malaria inflammation of the liver was characterized by degeneration of hepatocellular tissue, necrosis, and hyperplasia of Kupper cells [34]. Lung injury appeared as an alveolar hemorrhage [35], and kidney failure was associated with tubular hemoglobin casts [2,30]. Similar cytotoxicity in many organs was observed during the acute infection of all species of malaria such as *P. falciparum* and *P. vivax* [36,37]. On the other hand, the immunohistochemistry investigation of TNF-α and IL-6 expressed strong positive reactions with pulmonary components that reflected the severity of malaria damage and lung inflammation of *P. chabaudi*. Similar upregulation was displayed in inflammatory cytokines, IL-1β, IL-6, and TNF-α in the infected spleen with *P. chabaudi* [38,39]. There is a noticeable difference in the degree of intensity of TNF-α and IL-6 in lung tissue whereas IL-6 cytokine recorded a heavy stain than TNF-α in the same infected group. Yang et al. [40] and Tomkins-Netzer et al. [41] referred to these intensity variations to the different roles of each cytokine in the inflammatory response, while the TNF-α initiated the inflammatory response rapidly by triggering the immune cells to the inflammatory site; consequently, their expression was not prolonged for a long time. Additionally, Popko et al. [42] and Boulares et al. [43] revealed that IL-6 amplified the immune response, increased the vascular permeability, and was associated with the development of edema, therefore their prolonged expressions were registered.

Treatment with *I. oblongifolia* leaf extract and AgNPs resulted in rapid recovery from cytotoxicity in the lungs. Respiratory ducts restored to their usual size and thickness. Also, most histopathological signs like congestion, necrosis, and edema returned to their normal levels. The structural reorganization became more evident compared to the infected lung. Lubbad et al. [44] and Al-Quraishy et al. [45] referred this recovery to the anti-inflammatory and anti-oxidant properties of this medical extraction that were able to restore the normal organization of white and red pulps in the spleen after infection with *P. chabaudi.* Zhang et al. [46] and Khan et al. [47] explained that the antimalarial impact of novel therapy was represented in the disruption of the viability of the parasite through silver ion of AgNPs which in turn suppressed the mitochondrial electron transport chain, and stopped ATP synthesis in plasmodium. In addition to the previous findings Zahra et al. [48] referred to flavonoids of *I. oblongifolia* that acted on neutralizing the ROS and reducing lipid peroxidation. The anti-inflammatory properties of *I. oblongifolia* appeared in the suppression of NF-κB activation, which in turn reduced the overproduction of pro-inflammatory cytokines and preserved necessary immune responses according to Almayouf et al. [49]. Moreover, Abdel Moneim [50] and Destro et al. [51] showed the protective, anti-fibrotic, anti-oxidant, and anti-apoptotic activities of *I. oblongifolia* in restoring the normal organization of hepatocellular cells after hepatocytotoxicity of lead acetate. Furthermore, during the recovery by *I. oblongifolia*, the immunohistochemistry expression of TNF-α and IL-6 was low in comparison to infected tissue. Analogous expression was registered in cancerous mice treated with *I. trita* [52].

Conversely, applying CQ 10 mg/kg after inflammation with *P. chabaudi* as a malaria local medical drug did not achieve full recovery of pulmonary cytotoxicity in the present study. However, the bronchiolar and alveolar ducts still suffered from little obstruction and thicker walls than the normal ones. In addition, cytotoxic signs such as edema and necrosis were still observed with significant intensity in this lung. Alven and Aderibigbe [53] and Tiwari et al. [54] referred the low response of CQ as a conventional antimalarial drug to the molecular resistance of antimalarial drugs in several species of parasites which had polymorphisms in proteins that change the physiological regulation in the parasite. The standard antimalarial medication (CQ, tafenoquine, and primaquine) has a poor response because to its limited water solubility, bioavailability, and short half-life [55,56]. However, Zhou et al. [57] and Ravindar et al. [58] showed the antimalarial, anticancer, and antiviral response of CQ and considered it as a promising therapeutic potential in autoimmune diseases, metabolic disorders, cardiovascular diseases, and neurodegenerative diseases. Also, Chaves et al. [59] added that due to the increasing resistance of local medical drugs like CQ, lumefantrine, and primaquine, there is an urgent need for a diversity of treatment ways and application of nanoscale size of some medical plants to facilitate the penetration and adsorption of therapy. Another implication registered against CQ as an effective therapy for malaria disease was its limited efficiency in restoring normal lung architecture. Yang et al. [60] and Gong et al. [61] reported that CQ was unable to modulate the host inflammatory response and also could not be involved in tissue regeneration that assisted in lung recovery. On the other hand, this mixture of IOLE AgNPs had potent anti-oxidant and anti-inflammatory properties which reduced the oxidative stress status besides suppressing the pro-inflammatory cytokines post-infection, which was responsible for persistent inflammation that mediated lung damage according to Jangid et al. [62] and Parvin et al. [63] who confirmed that the nano-particles modulated chemokines and growth factors like G-CSF and CXCL1 for tissue repair.

The significant coordination in the expression of TNF-α and IL-6 that appeared through the heat map of Pearson correlation demonstrated the well-inflammatory response in the infected tissues with *P. chabaudi* that was registered in the form of edema, obstruction of pulmonary ducts, and congestion. Also, this coordination was significantly observed in the decrease in their expression after the treatment with IOLE AgNPs which was recorded in the form of withdrawal of majority of histopathological signs from the tissue. Comparable findings were demonstrated in Bhol et al. [64] who showed that curcumin was able to down-regulate the levels of proinflammatory cytokines of TNF-α and IL-6 in various models of acute inflammation. Moreover, Mrityunjaya et al. [65] and Carlson et al. [66] showed that a significant reduction in these levels of cytokines led to the withdrawal of the histopathological signs from the tissues.

## 7 Conclusion

In conclusion, the accumulation of AgNPs triggered proinflammatory cytokines and created an oxidative stress status in lung tissue. Therefore, *I. oblongifolia* as a natural medical plant displayed significant antimalarial and anti-oxidant properties effectively reducing inflammatory signs and cytokine levels in *P. chabaudi-*infected lungs and the harmful impact of AgNPs in *P. chabaudi-*infected + *I. oblongifolia* with AgNPs, while CQ (Q 10) shows limited efficiency due to molecular drug resistance. This study highlights the therapeutic potential of *I. oblongifolia* for malaria management.
